# Limiting the Use of Oral Glucose Tolerance Tests to Screen for Hyperglycemia in Pregnancy during Pandemics

**DOI:** 10.3390/jcm10030397

**Published:** 2021-01-21

**Authors:** Charlotte Nachtergaele, Eric Vicaut, Sopio Tatulashvili, Sara Pinto, Hélène Bihan, Meriem Sal, Narimane Berkane, Lucie Allard, Camille Baudry, Jean-Jacques Portal, Lionel Carbillon, Emmanuel Cosson

**Affiliations:** 1 AP-HP, Unité de Recherche Clinique St-Louis-Lariboisière, Université Denis Diderot, 75009 Paris, France; nachtergaele.charlotte@yahoo.fr (C.N.); eric.vicaut@aphp.fr (E.V.); jean-jacques.portal@aphp.fr (J.-J.P.); 2 AP-HP, Department of Endocrinology-Diabetology-Nutrition, Avicenne Hospital, Paris 13 University, Sorbonne Paris Cité, CRNH-IdF, CINFO, 93 000 Bobigny, France; sopio.tatulashvili@aphp.fr (S.T.); helene.bihan@aphp.fr (H.B.); meriem.sal@aphp.fr (M.S.); narimane.berkane@aphp.fr (N.B.); lucie.allard@aphp.fr (L.A.); camille.baudry@aphp.fr (C.B.); 3 AP-HP, Department of Endocrinology-Diabetology-Nutrition, Jean Verdier Hospital, Paris 13 University, Sorbonne Paris Cité, CRNH-IdF, CINFO, 93 143 Bondy, France; sara.pinto@aphp.fr; 4 AP-HP, Department of Obstetrics and Gynecology, Jean Verdier Hospital, Paris 13 University, Sorbonne Paris Cité, 93 000 Bondy, France; lionel.carbillon@aphp.fr; 5 Paris 13 University, Sorbonne Paris Cité, UMR U557 INSERM/U11125 INRAE/CNAM/Université Paris13, Unité de Recherche Epidémiologique Nutritionnelle, 93 000 Bobigny, France

**Keywords:** Australian Diabetes in Pregnancy Society (ADIPS), Australian Diabetes Society (ADS), COVID-19, gestational diabetes mellitus, oral glucose tolerance test, pandemic, hyperglycemia in pregnancy, pregnancy outcomes, Royal Australian and New Zealand College of Obstetricians and Gynaecologists (RANZOG)

## Abstract

We aimed to evaluate each proposal of Australian–New Zealand Societies to limit the number of oral glucose tolerance tests (OGTTs) to diagnose hyperglycemia in pregnancy (HIP) during the coronavirus disease 2019 (COVID-19) pandemic. At our university hospital (2012–2016), we retrospectively applied in 4245 women who had OGTT between 22 and 30 weeks of gestation (reference standard: WHO criteria) the proposals in which OGTT is performed only in high-risk women; in all (Option 1) or high-risk (Option 1-Sel) women with fasting plasma glucose (FPG) 4.7–5.0 mmol/L; in all (Option 2) or high-risk (Option 2-Sel) women without history of HIP and with FPG 4.7–5.0 mmol/L. We also tested FPG measurement alone in all high-risk women. Measuring FPG alone had a sensitivity of 49% (95% confidence interval 45–54) applying universal screening. Option 2 appeared to have the best balance considering the needed OGTT (17.3%), sensitivity (72% (67–76)) and rates of a composite outcome (true negative cases: 10.6%, false positive cases: 24.4%; true positive cases: 19.5%; false negative cases: 10.2%). Consideration of a history of HIP and measuring first FPG can avoid more than 80% of OGTTs and identify women with the highest risk of adverse HIP-related events.

## 1. Introduction

Hyperglycemia in pregnancy (HIP) refers to gestational diabetes mellitus (GDM) and diabetes in pregnancy (DIP) [1,2,3,4,5,6]. DIP is considered as unknown pregravid diabetes and is usually screened using fasting plasma glucose (FPG) or HbA1c measurement in early pregnancy. DIP is associated with increased risk of stillbirth rate [7]. Early-diagnosed HIP is usually immediately treated. If early screening is normal, a new screening is performed in the late second trimester or early third trimester. Diagnosis is based on the oral glucose tolerance test (OGTT, the reference standard), with measurements of FPG, and one-hour (1h-PG), two-hour (2h-PG) and sometimes three-hour plasma glucose [1,2,3,4,5,6]. Identifying and treating HIP diagnosed at that time reduces maternal and neonatal events [8,9].

Considering the current coronavirus disease 2019 (COVID-19) pandemic, pregnant women are advised to be stringent with public health measures such as social distancing and self-isolation to lower their risk of exposure. However, OGTT measurements require long times spent at OGTT testing centers. Therefore, temporary changes to the process of diagnostic testing for HIP need to be considered [10]. As proposed in Australia and New Zealand [11,12,13], such a perspective is to reduce the percentage of women who need to undergo an OGTT, whereby OGTTs may be indicated only in women with intermediate FPG values [11]. OGTTs may also be avoided in women with history of HIP who would be considered to have current HIP [11]. HIP might also be based on FPG measurement alone [12]. Finally, selective rather than universal screening could be applied. However, a poor sensitivity of such strategies could be deleterious, because unidentified women with HIP would not be managed. On the contrary, a poor specificity could lead to care for women without HIP.

We had the opportunity in our large retrospective cohort of women [14,15] to evaluate for seven options: (i) the percentage of women who would be selected to undergo OGTTs if these proposals would have been applied; (ii) the percentage of HIP who would have been diagnosed or not; and (iii) the occurrence of adverse outcomes if the women would have been correctly diagnosed or not, with a special interest for false negative and false positive cases of HIP.

## 2. Materials and Methods

### 2.1. Data Collection

This observational study was conducted in our university hospital in a suburban area of Paris, France, and was based on routine electronic medical records of maternal and neonatal events at birth between January 2012 and October 2016 [14,15]. In addition, we have collected data on HIP screening in all women. Women were informed that their medical records could be used for research, unless they opposed [14,15]. The data were analyzed anonymously. Our database was declared to the French Committee for computerized data (CNIL: Commission Nationale de l’Informatique et des Libertés, number 1704392v0).

### 2.2. Screening for Hyperglycemia in Pregnancy

We follow the French recommendations in our center [3], except that our policy is to universally screen women, both at the beginning of pregnancy and after 24 weeks of gestation (WG) if prior screening was normal or not done. Early screening during pregnancy is based on FPG measurement. Women with FPG levels ≥5.1 mmol/L are diagnosed with HIP. Those without early-diagnosed HIP undergo a 75 g OGTT between 24 and 28 WG, with measurements of FPG, 1h-PG and 2h-PG [3]. The International Association of Diabetes Pregnancy Study Group (IADPSG) [1] and World Health Organization (WHO) recommendations [2] are considered for HIP diagnosis, because these guidelines have been endorsed in France [3]. Accordingly, GDM was defined as FPG 5.1–6.9 mmol/L and/or 1h-PG ≥ 10.0 mmol/L and/or 2h-PG 8.5–11.0 mmol/L in the OGTT, whereas DIP was defined as FPG ≥ 7.0 and/or 2h-PG ≥ 11.1 mmol/L.

### 2.3. Selection Criteria for Our Study

Inclusion criteria were woman who had an OGTT between 22 and 30 WG, were 18 to 50 years old, single fetus pregnancies, and had no personal history of either diabetes or bariatric surgery. We considered OGTT results between 22 and 30 WG rather than between 24 and 28 WG because OGTTs were often used during this period of time [14]. We then selected the women whose risk factor for HIP status was known, and applied Australian–New Zealand risk factors [11]. They include any of the following factors: previous history of HIP or neonatal death; previously elevated blood glucose level (not available in our data set (NA)); maternal age ≥40 years; family history of diabetes; pre-pregnancy obesity (body mass index > 30 kg/m^2^); previous baby with macrosomia; polycystic ovarian syndrome (NA); corticosteroids and antipsychotics medication (NA) and finally ethnicity. We have previously shown that North African, Indian, Pakistani, Sri Lankan, and Asian ethnicities were at high risk in our cohort [16]. We finally selected women who had no HIP in early pregnancy, defined as FPG levels <5.1 mmol/L (Flow chart in Figure S1).

### 2.4. Description of Tested Algorithms

**Figure 1:**
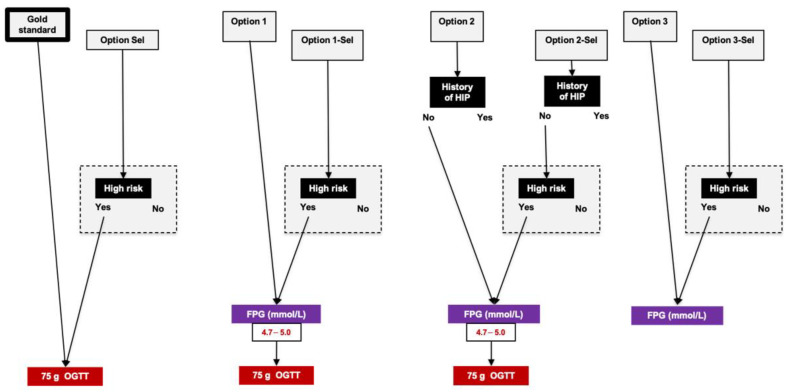
Reference standard and options that were evaluated after 22 weeks of gestation. Reference standard refers to universal screening with oral glucose tolerance test between 22 and 30 weeks of gestation (75 g oral glucose tolerance test, IADPSG/WHO criteria). We evaluated seven options, applying universal (Options 1, 2 and 3) or selective screening (Options Sel, 1-Sel, 2-Sel and 3-Sel). Women were considered at high risk according to Australian–New Zealand risk factors (please see text). OGTT: oral glucose tolerance test; 1h-PG and 2h-PG: plasma glucose value 1 and 2 h after 75 g oral glucose tolerance test, respectively; DIP: diabetes in pregnancy; FPG: fasting plasma glucose; GDM: gestational diabetes mellitus; HIP: hyperglycemia in pregnancy.

The reference standard testing was the results of OGTTs between 22 and 30 WG according to IADPSG/WHO criteria applying universal screening. Figure 1 shows the seven tested algorithms (termed as “Options”) in which:

- OGTTs would be performed only in women with risk factor for HIP, i.e., applying selective screening (Option Sel);
- OGTTs would be performed in women with FPG 4.7–5.0 mmol/L between 22 and 30 WG, applying universal (Option 1) or selective screening (Option 1-Sel) [11];
- OGTTs would be performed in women without history of HIP (those with previous HIP are considered to have GDM) and with FPG 4.7–5.0 mmol/L between 22 and 30 WG, applying universal (Option 2) or selective screening (Option 2-Sel) [11];
- FPG alone would be measured, applying universal (Option 3) or selective screening (Option 3-Sel) [12].

If the new proposals were applied, the women would be classified as:

- True negative: women who have no HIP (IADPSG/WHO criteria, universal screening) and would be correctly diagnosed as having no HIP with the tested proposal;
- False positive: women who have no HIP (IADPSG/WHO criteria, universal screening) but would be diagnosed as having HIP with the tested proposal;
- True positive: women who have HIP (IADPSG/WHO criteria, universal screening) and would be correctly diagnosed as having HIP with the tested proposal;
- False negative: women who have HIP (IADPSG/WHO criteria, universal screening) but would be misdiagnosed with the tested proposal.

### 2.5. Adverse Outcomes

The main predefined endpoint was the occurrence of a composite adverse outcome, which included at least one of the following events: (i) preeclampsia (blood pressure ≥ 140/90 mmHg on two recordings four hours apart and proteinuria of at least 300 mg/24 h or 3+ on dipstick testing in a random urine sample); (ii) an infant large for the gestational age (LGA: birth weight greater than the 90th percentile for a standard French population [14,15]); (iii) shoulder dystocia, defined as the use of obstetrical maneuvers (McRoberts maneuver, episiotomy after delivery of the fetal head, suprapubic pressure, posterior arm rotation to an oblique angle, rotation of the infant by 180 degrees, or delivery of the posterior arm); and (iv) neonatal hypoglycemia, defined as at least one blood glucose value less than 36 mg/dL during the first two days of life [14,15].

We also considered each of the previous events separately, and additionally infants small for the gestational age (birth weight lower than the 10th percentile for a standard French population [14,15]; selective and emergency (before or during delivery) caesarean sections; preterm delivery (delivery before 37 completed weeks); admission to a neonatal intensive care unit; respiratory distress syndrome (based on the clinical course, chest X-ray findings, blood gas and acid–base values); and finally intrauterine fetal or neonatal death (in the first 24 h of life). We also considered the need for insulin at the time of delivery [17].

To note, all women with HIP were referred to our multidisciplinary team including a diabetologist, an obstetrician, a midwife, a dietician, and a nurse educator, and managed according to French recommendations. They received individualized dietary advice, instructions on how to perform self-monitoring of blood glucose levels six times a day, and were seen by the diabetologist every 2–4 weeks. They received insulin therapy when pre-prandial and 2 h post-prandial glucose levels were greater than 5.0 and 6.7 mmol/L respectively, according to the French guidelines [3]. Obstetrical care also followed the French recommendations [3]. Timing and mode of delivery was discussed with the patient and obstetrical staff according to fetal weight estimation during ultrasound scans at 37 WG and considering glucose control. At 39 WG, labor induction (using prostaglandin E2 or oxytocin infusion) or even caesarean section was possibly decided according to obstetric history, maternal condition, and estimated fetal weight. Continuous fetal cardiotocography was routinely used during labor. Overall, it must be considered that, in our cohort, false negative cases of HIP were cared for, whereas false positive cases were not.

### 2.6. Statistics

Baseline continuous variables were expressed as the mean ± standard deviation (SD). Categorical variables were expressed as frequencies (percentages). First, we evaluated the proportions of women selected to undergo OGTTs according to each screening option. We then evaluated the performance of each option for screening for HIP after 22 WG. The reference standard was the results of the OGTT between 22 and 30 WG, applying universal screening. We considered sensitivity, specificity, positive (PPV), and negative predictive value (NPV) of each option.

We therefore compared characteristics and adverse outcome rates of true negative, false positive, true positive and false negative cases of HIP according to each option. To compare continuous variables by the different groups of patients (True negative, False positive, True positive and False negative), we used ANOVA. To compare categorical variables, we used the Chi-squared (*χ*^2^) test or Fisher’s exact test. Values were considered significant at a probability level of 0.05. For the difference between each group of patients by each other, we performed a post-hoc analysis for multiplicity by Bonferroni method and adjusted the *p*-value depending on the number of tests made in each option evaluated. All tests were two-sided. Analyses were conducted using R 3.6.3 software (R Foundation, Vienna, Austria, https://cran.r-project.org).

## 3. Results

### 3.1. Population Characteristics

We included 4245 women (Flow chart in Figure S1). Their baseline characteristics are shown in Table 1.

### 3.2. Limiting the Percentage of Women Who Undergo OGTTs

The percentage of women who would have had OGTTs in our series was the highest for Option Sel (48.3%), then progressively decreased from Option 1 (18.5%), to Option 2 (17.3%), Option 1-Sel (9.7%) then Option 2-Sel (8.5%). There were no OGTTs performed for Options 3 and 3-Sel (Table 2).

### 3.3. Performance of Each Option to Diagnose HIP Cases

Table 2 shows the sensitivity, specificity, PPV and NPV of each option. Globally, sensitivities were around 70% for Option Sel, Options 1 and 2; around 50% for Options 1-Sel, 2-Sel and 3; and 33% for Option 3-Sel. Specificities were 98–100% for all options. PPV was 100% for all options (meaning there were no false positive cases), except for Option 2 and 2-Sel. In Option 2, the PPV was 80% (76–97%) and in Option 2-Sel, it was 73% (67–78%). Finally, NPV was higher than 90% for all options.

### 3.4. Characteristics of True Negative, False Positive, True Positive of False Negative Cases of HIP, and Their Prognosis

True/false negative/positive cases of HIP defined by each option are compared in a specific table by option: Option Sel (Supplementary Table S1), Option 1 (Supplementary Table S2), Option 1-Sel (Supplementary Table S3), Option 2 (Table 1), Option 2-Sel (Supplementary Table S4), Option 3 (Supplementary Table S5), Option 3-Sel (Supplementary Table S6). We chose to especially show results for Option 2, because this option appeared to have the best balance between the reduction in the percentage of OGTTs (by 82.7%) and identification of the women with the highest risk of adverse outcomes.

Globally, false negative cases as compared to true positive cases of HIP had a significantly lower preconception body mass index, with statistically different glucose values during the OGTTs. Additionally, in the options where selective screening was applied, false negative cases had fewer risk factors than true positive cases.

For Option 2 and 2-Sel, false positive cases had a personal history of HIP. As compared to true negative cases, they had higher FPG, 1h-PG, and 2h-PG values during OGTTs, were older, and were more prone to have, in addition to personal history of HIP, personal history of macrosomic infants or a family history of diabetes.

### 3.5. Prognosis Associated with True Negative, False Positive, True Positive and False Negative Cases of HIP

**Figure 2:**
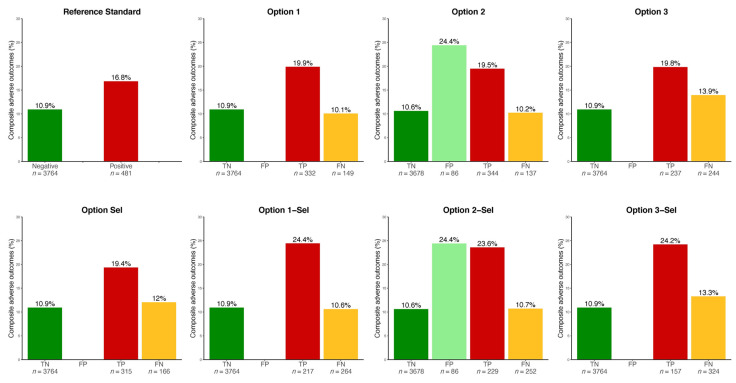
Rate of hyperglycemia in pregnancy-related events by true/false positive/negative cases by each option. Please see description of each option in Figure 1. TN, FP, TP, FN: true negative, false positive, true positive, false negative of hyperglycemia in pregnancy, respectively. HIP-related events: hyperglycemia in pregnancy-related events (composite: preeclampsia or large for gestational age infant or shoulder dystocia or neonatal hypoglycemia). Note that TP and FN cases had been treated for HIP in this observational cohort, whereas TN and FP cases had not.

The same tables and Figure 2 show the rate of the composite adverse outcome in each group. Especially, the false negative cases, as compared to the true positive cases of HIP, had fewer adverse events during pregnancy—especially HIP-related events, LGA infants, and neonatal hypoglycemia.

Finally, the false positive cases as compared to the true negative cases (Option 2 and Option 2-Sel only) had significantly more composite adverse outcomes. This was driven by a higher rate of LGA infants.

## 4. Discussion

In the current study, we compared the diagnostic performance of various screening strategies for HIP diagnosis and to identify the women more prone to experience HIP-related events, with the aim to prevent a large proportion of pregnant women from undergoing an OGTT. This is particularly crucial during the COVID-19 pandemic. One option (Option 2) appears to offer a good compromise because it reduces the rate of women undergoing OGTTs by more than 80%, while it identifies around 70% of the women with HIP, especially those (both false positive and true positive cases) with the highest risk of adverse outcomes.

The sensitivity of selective rather than universal screening to identify women with HIP defined according to the IADPSG/WHO criteria has been shown to be between 60% and 95% [15,18,19,20,21]. We show here that performing OGTTs only in high-risk women had a sensitivity of 65%. In our series, 48.3% of women would have had an OGTT if selective screening had been applied. This rate would depend on locally considered risk factors and their prevalence. Whatever the options, a selective policy led to reduction by around one-half of the rate of screening. Using alternative options would lead to preventing more women from performing OGTTs.

In this perspective, several strategies have been suggested. Some include HbA1c measurement [11,22]. However, the use of HbA1c for diagnosing HIP has been disappointing because there is substantial overlap between women with normoglycemia and women with HIP. This has particularly been shown for HIP defined according to IADPSG/WHO criteria [23,24,25,26,27,28,29]. Some other strategies include a single random glucose measurement [30,31]. However this was considered to be inadequate to screen for HIP in a systematic review [32], due to its low sensitivity [33].

As shown in our study, FPG measurement alone is also not highly sensitive [34,35,36]. For example, in the Hyperglycemia Adverse Outcomes in Pregnancy (HAPO) study, one-half of GDM cases were detected through elevated 1h- and/or 2h-PG, whereas FPG levels were normal [34]. However, studies using IADPSG/WHO criteria showed FPG to be useful for simplifying the screening process and reducing the number of OGTTs [34,35,36]. Indeed, FPG thresholds of ≤4.4 mmol/L have been reported to rule out HIP in 50–65% of women with a sensitivity of 80–95% [34,35,36,37]. As proposed by one guidance [11], we used a FPG level <4.7 mmol/L to rule out women with HIP in this series. Sensitivities of such options were around 70% applying universal screening and 45% applying selective screening, which appears imperfect.

According to Australian–New Zealand guidance [11], women with a history of HIP might be considered as presenting current GDM (Options 2 and 2-Sel). In fact, as previously reviewed [38], the HIP recurrence rate is around 50%, and therefore leads to false positive cases of HIP. Over-management of these women might induce infants small for the gestational age, and will result in more testing, monitoring and contact with hospital services throughout their pregnancy. Similarly, not caring for women with HIP because they are not diagnosed might also be associated with adverse outcomes [8,9]. We therefore compared prognoses associated with false positive vs true negative cases of HIP and with false negative vs true positive cases.

We report for the first time that false positive cases of HIP, i.e., women with a history of HIP but a normal OGTT (Options 2 and 2-Sel), had a worse prognosis than true negative cases. One explanation is that: (i) they had higher glucose levels during OGTTs which correlates with more adverse outcomes [34]; and (ii) they had more risk factors for HIP, including history of personal HIP, also associated with a poor prognosis [15]. On the one hand, this suggests that management of these women with diet, exercise and possibly insulin treatment might be useful to reduce the number of HIP-related events. On the other hand, this implies more contact with hospital services throughout pregnancy.

Additionally, we have shown that the prognosis of false negative cases was better than that for true positive cases, but this is only partly reassuring. Indeed, these cases were actually managed for HIP, as observed in this study. Moreover, around one-quarter of false negative cases of HIP were insulin-treated in our series. One retrospective study based on the HAPO data suggested that missed diagnosed GDM with the COVID-19 proposal could present fewer events than those who are not missed, even when they are not treated [39]. This especially completes our data because the women included in the HAPO study were not treated. Overall, we do not know what the loss of treatment benefit would be if these women had not been managed, and randomized studies would be necessary to draw definitive conclusions. Indeed, not caring for HIP in low-risk women might lead to a doubling of the rate of adverse events during pregnancy [8,9].

The strengths of our study include the large numbers of subjects and a multiethnic cohort likely to be translatable to different populations, and a pragmatic guidance-based approach. The prospectively collected standardized data provide for a robust investigational data set and we could investigate several options in the same series of women. We excluded women who had no FPG measurements or had FPG levels ≥5.1 mmol/L in early pregnancy, whereas some guidance proposes to screen for HIP in early pregnancy with random plasma glucose and/or HbA1c measurement [11]. We limited our evaluation for women who underwent OGTTs in the late second and early third trimesters (22–30 WG). We could not consider preanalytical issues for FPG measurement: the time interval between sampling and spinning fasting glucose measurements can double or half the diagnoses of GDM [40]. Finally, an additional strength was the evaluation of the prognosis of true/false negative/positive cases, although while interpreting the results, we had to consider that false negative cases were managed for HIP in our series.

## 5. Conclusions

To conclude, during current and future pandemics, consideration that every pregnant woman should undergo an OGTT at the end of the second trimester is an important issue. We show here that FPG measurement first can avoid 80–90% of OGTTs. The sensitivity of such an option is around 70% and 50% applying universal and selective screening, respectively. In both cases, however, the women at the highest risk of adverse HIP-related events during pregnancy are identified and therefore would be managed during pregnancy. Any changes to international guidelines before the pandemic could be replaced by some options tested in this study only temporarily, pending resolution of the COVID-19 pandemic, and in any case with the awareness of exposing some women with HIP to the risk of not being identified and therefore not being treated. However, such a screening regimen may be applicable in countries where OGTTs are difficult to perform.

## Figures and Tables

**Table 1:** Characteristics of the women by true/false positive/negative cases considering Option 2.

	Total	True Negative Cases	False Positive Cases	True Positive Cases	False Negative Cases	*p*
	*n* = 4245	*n* = 3678	*n* = 86	*n* = 344	*n* = 137	
OGTT between 22 and 30 WG						
Fasting plasma glucose (mmol/L)	4.38 (0.45)	4.30 (0.36) ^a,b^	4.46 (0.37) ^d,e^	5.23 (0.47) ^f^	4.29 (0.26)	<0.001
1-h plasma glucose (mmol/L)	6.76 (1.76)	6.42 (1.46) ^a,b,c^	7.48 (1.42) ^d,e^	9.17 (2.02) ^f^	9.61 (1.24)	<0.001
2-h plasma glucose (mmol/L)	5.96 (1.43)	5.67 (1.10) ^a,b,c^	6.34 (1.20) ^d,e^	7.93 (1.93) ^f^	8.58 (1.34)	<0.001
Gestational age at time of OGTT (WG)	26.22 (1.89)	26.21 (1.88)	26.19 (2.03)	26.29 (1.91)	26.40 (1.85)	NS
Characteristics						
Age (years)	30.25 (5.32)	29.93 (5.25) ^a,b,c^	32.38 (4.74)	32.42 (5.28)	32.01 (5.60)	<0.001
Preconception body mass index (kg/m^2^)	24.36 (4.48)	24.15 (4.36) ^b^	25.31 (4.55)	26.30 (5.14) ^f^	24.57 (4.46)	<0.001
Obesity	493 (11.7)	388 (10.6) ^b^	14 (16.7)	76 (22.2) ^f^	15 (10.9)	<0.001
Preconception hypertension	28 (0.7)	19 (0.5) ^b^	1 (1.2)	7 (2.0)	1 (0.7)	0.01
Family history of diabetes	824 (19.4)	671 (18.2) ^a,b^	28 (32.6)	94 (27.3)	31 (22.6)	<0.001
Employment	1883 (44.4)	1649 (44.9)	28 (32.6)	148 (43.1)	58 (42.3)	NS
Smoking before pregnancy	493 (11.6)	447 (12.2)	3 (3.5)	34 (9.9)	9 (6.6)	0.012
Parity	2.03 (1.18)	2.00 (1.17)	2.90 (1.05)	2.28 (1.23)	1.83 (1.12)	
Previous pregnancy(ies)						
History of hyperglycemia in pregnancy						<0.001 *
First child	1769 (41.7)	1589 (43.2)	0 (0.0)	108 (31.4)	72 (52.6)	
No	2324 (54.7)	2089 (56.8)	0 (0.0)	170 (49.4)	65 (47.4)	
Yes	152 (3.6)	0 (0.0) ^a,b^	86 (100.0) ^d,e^	66 (19.2) ^f^	0 (0.0)	
History of macrosomia						<0.001 *
First child	1769 (41.7)	1589 (43.2)	0 (0.0)	108 (31.4)	72 (52.6)	
No	2378 (56.0)	2022 (55.0)	77 (89.5)	218 (63.4)	61 (44.5)	
Yes	98 (2.3)	67 (1.8) ^a,b^	9 (10.5)	18 (5.2)	4 (2.9)	
History of hypertensive disorders						NS *
First pregnancy	1226 (28.9)	1108 (30.1)	0 (0.0)	68 (19.8)	50 (36.5)	
No	2941 (69.3)	2504 (68.1)	84 (97.7)	268 (77.9)	85 (62.0)	
Yes	78 (1.8)	66 (1.8)	2 (2.3)	8 (2.3)	2 (1.5)	
History of fetal death						0.04 *
First pregnancy	1226 (28.9)	1108 (30.1)	0 (0.0)	68 (19.8)	50 (36.5)	
No	2964 (69.8)	2528 (68.7)	84 (97.7)	266 (77.3)	86 (62.8)	
Yes	55 (1.3)	42 (1.1) ^b^	2 (2.3)	10 (2.9)	1 (0.7)	
Ethnicity						<0.01
North African	866 (20.4)	694 (18.9)	29 (33.7)	108 (31.5)	35 (25.5)	
European	1509 (35.6)	1353 (36.8)	16 (18.6)	93 (27.1)	47 (34.3)	
Sub-Saharan African	888 (20.9)	793 (21.6)	15 (17.4)	69 (20.1)	11 (8.0)	
Indian-Pakistan-Sri Lankan	342 (8.1)	267 (7.3)	15 (17.4)	44 (12.8)	16 (11.7)	
Caribbean	281 (6.6)	260 (7.1)	3 (3.5)	13 (3.8)	5 (3.6)	
Asian	72 (1.7)	59 (1.6)	2 (2.3)	3 (0.9)	8 (5.8)	
Other	285 (6.7)	251 (6.8)	6 (7.0)	13 (3.8)	15 (10.9)	
High-risk women	2050 (48.3)	1649 (44.8)	86 (100.0)	229 (66.6)	86 (62.8)	
Glycemic status (reference standard: IADPSG/WHO criteria)						<0.001
Normal	3764 (88.7)	3678 (100.0)	86 (100.0)	0 (0.0)	0 (0.0)	
Gestational diabetes mellitus	459 (10.8)	0 (0.0)	0 (0.0)	326 (94.8)	133 (97.1)	
Diabetes in pregnancy	22 (0.5)	0 (0.0)	0 (0.0)	18 (5.2)	4 (2.9)	
Events during pregnancy						
Composite adverse outcome	492 (11.6)	390 (10.6) ^a,b^	21 (24.4) ^e^	67 (19.5)	14 (10.2)	<0.001
Preeclampsia	71 (1.7)	59 (1.6)	1 (1.2)	6 (1.7)	5 (3.6)	0.29
LGA age infant	400 (9.4)	318 (8.6) ^a,b^	20 (23.3) ^e^	54 (15.7) ^f^	8 (5.8)	<0.001
Shoulder dystocia	6 (0.1)	4 (0.1)	1 (1.2)	0 (0.0)	1 (0.7)	0.06
Neonatal hypoglycemia	27 (0.6)	15 (0.4) ^b^	0 (0.0)	11 (3.2)	1 (0.7)	<0.001
Ceasarean section	862 (20.3)	721 (19.6) ^b^	23 (26.7)	90 (26.2)	28 (20.4)	0.014
Preterm delivery (<37 weeks)	229 (5.4)	193 (5.2)	3 (3.5)	24 (7.0)	9 (6.6)	0.42
Offspring hospitalization	812 (19.1)	677 (18.4)	21 (24.4)	81 (23.5)	33 (24.1)	0.026
Respiratory distress syndrome	202 (4.8)	166 (4.5)	7 (8.1)	18 (5.2)	11 (8.0)	0.11
Intrauterine fetal or neonatal death	13 (0.3)	11 (0.3)	1 (1.2)	1 (0.3)	0 (0.0)	0.39
SGA infant	417 (9.8)	366 (10.0)	2 (2.3) ^e^	30 (8.7)	19 (13.9)	0.04
Insulin therapy during	172 (4.1)	0 (0.0) ^b,c^	0 (0.0) ^d,e^	140 (40.7) ^f^	32 (23.4)	<0.001

Date are *n* (%) or mean (standard deviation). HIP: hyperglycemia in pregnancy; IADPSG: International Association of Diabetes Pregnancy Study Group; LGA: large for gestational age; OGTT: oral glucose tolerance test; SGA: small for gestational age; WG: weeks of gestation; WHO: World Health Organization. Composite adverse outcome: preeclampsia or LGA infant or shoulder dystocia or neonatal hypoglycemia. Symbols indicate whether values are significant (*p* < 0.05) after Bonferroni adjustment for multiplicity: ^a^ True negative versus False positive, ^b^ True negative versus True positive, ^c^ True negative versus False negative, ^d^ False positive versus True positive, ^e^ False positive versus False negative, ^f^ True positive versus False negative; * yes versus no comparison; NS: non-significant.

**Table 2:** Percentage of women who underwent oral glucose tolerance test and performance of to diagnose hyperglycemia in pregnancy, by each option.

	Number of OGTTs	Sensitivity	Specificity	PPV	NPV
Option Sel	2050 (48.3)	0.65 (0.61–0.70)	1.00 (1.00–1.00)	1.00 (0.99–1.00)	0.96 (0.95–0.96)
Option 1	786 (18.5)	0.69 (0.65–0.73)	1.00 (1.00–1.00)	1.00 (0.99–1.00)	0.96 (0.96–0.97)
Option 1-Sel	413 (9.7)	0.45 (0.41–0.50)	1.00 (1.00–1.00)	1.00 (0.98–1.00)	0.93 (0.93–0.94)
Option 2	735 (17.3)	0.72 (0.67–0.76)	0.98 (0.97–0.98)	0.80 (0.76–0.97)	0.96 (0.96–0.97)
Option 2-Sel	362 (8.5)	0.48 (0.43–0.52)	0.98 (0.97–0.98)	0.73 (0.67–0.78)	0.94 (0.93–0.97)
Option 3	0	0.49 (0.45–0.54)	1.00 (1.00–1.00)	1.00 (0.98–1.00)	0.94 (50.93–0.95)
Option 3-Sel	0	0.33 (0.28–0.37)	1.00 (1.00–1.00)	1.00 (0.98–1.00)	0.92 (0.91–0.93)

Data are *n* (%) or unit (95% confidence interval). NPV: negative predictive value; OGTT: oral glucose tolerance test; PPV: positive predictive value.

## Data Availability

The datasets generated during and/or analyzed during the current study are not publicly available but are available from the corresponding author on reasonable request.

## Supplementary Materials

The following are available online at https://www.mdpi.com/2077-0383/10/3/397/s1. Table S1: Characteristics of the women by true/false positive/negative cases considering Option Sel; Table S2: Characteristics of the women by true/false positive/negative cases considering Option 1; Table S3: Characteristics of the women by true/false positive/negative cases considering Option 1-Sel; Table S4: Characteristics of the women by true/false positive/negative cases considering Option 2-Sel; Table S5: Characteristics of the women by true/false positive/negative cases considering Option 3; Table S6: Characteristics of the women by true/false positive/negative cases considering Option 3-Sel; Figure S1: Flow chart of the study.

## Abbreviations

OGTT: 75 g oral glucose tolerance test; 1h-PG: plasma glucose value 1 h after 75 g oral glucose tolerance test; 2h-PG: plasma glucose value 1 h after 75 g oral glucose tolerance test; BMI: body mass index; DIP: diabetes in pregnancy; FPG: fasting plasma glucose; GDM: gestational diabetes mellitus; HIP: hyperglycemia in pregnancy; IADPSG: International Association of Diabetes Pregnancy Study Group; NA: not available in our dataset; NPP: negative predictive value; PPV: positive predictive value; WG: weeks of gestation; WHO: World Health Organization.
