# Supplementary material for: Limiting the Use of Oral Glucose Tolerance Tests to Screen for Hyperglycemia in Pregnancy during Pandemics
Source: J Clin Med. 2021 Jan 21;10(3):397. doi: 10.3390/jcm10030397 (PMC7864504; doi:10.3390/jcm10030397)
Supplement: Supplementary file 1 [file jcm-10-00397-s001.pdf]

Additional Table S1: Characteristics of the women by true/false positive/negative cases considering Option Sel

	True negative cases	True positive cases	False negative cases	p
	n=3764	n=315	n=166	
**OGTT between 22 and 30 WG**				
Fasting plasma glucose (mmol/L)	4.30*† (0.36)	4.94 (0.58)	5.00 (0.63)	<0.001
1-hour plasma glucose (mmol/L)	6.44*† (1.46)	9.47‡ (1.78)	8.95 (1.90)	<0.001
2-hour plasma glucose (mmol/L)	5.68 *† (1.11)	8.21 (1.83)	7.93 (1.75)	<0.001
Gestational age when OGTT (WG)	26.21 (1.88)	26.27 (1.86)	26.42 (1.95)	NS
**Characteristics**				
Age (years)	29.99 *(5.25)	33.28 ‡ (5.39)	30.46 (4.84)	<0.001
Preconception body mass index (kg/m^2)	24.18 * (4.37)	26.85 ‡ (5.50)	23.83 (3.09)	<0.001
Preconception hypertension	20 (0.5)	5 (1.6)	3 (1.8)	0.014
Family history of diabetes	699 (18.6) *†	125 ‡ (39.7)	0 (0.0)	<0.001
Employment	1677 (44.6)	124 (39.4)	82 (49.7)	NS
Smoking before pregnancy	450 (12.0) *	16‡ (5.1)	27 (16.3)	<0.001
Parity	2.02* (1.18)	2.30‡ (1.23)	1.89 (1.15)	<0.001
**Previous pregnancy(ies)**				
History of hyperglycemia in pregnancy				<0.001§
First child	1589 (42.2)	97 (30.8)	83 (50.0)	
No	2089 (55.5)	152 (48.3)	83 (50.0)	
Yes	86 (2.3) *	66 (21.0) ‡	0 (0.0)	
History of macrosomia				<0.001§
First child	1589 (42.2)	97 (30.8)	83 (50.0)	
No	2099 (55.8)	196 (62.2)	83 (50.0)	
Yes	76 (2.0) *	22 (7.0) ‡	0 (0.0)	
History of hypertensive disorders				NS§
First pregnancy	1108 (29.4)	67 (21.3)	51 (30.7)	
No	2588 (68.8)	241 (76.5)	112 (67.5)	
Yes	68 (1.8)	7 (2.2)	3 (1.8)	
History of fetal death				0.003§
First pregnancy	1108 (29.4)	67 (21.3)	51 (30.7)	
No	2612 (69.4)	237 (75.2)	115 (69.3)	
Yes	44 (1.2)*	11 (3.5)	0 (0.0)	
**Ethnicity**				<0.001
North African	723 (19.2)	143 (45.4)	0 (0.0)	
European	1369 (36.4)	51 (16.2)	89 (53.9)	
Sub-Saharan African	808 (21.5)	21 (6.7)	59 (35.8)	
Indian-Pakistan-Sri Lankan	282 (7.5)	60 (19.0)	0 (0.0)	
Caribbean	263 (7.0)	13 (4.1)	5 (3.0)	
Asian	61 (1.6)	11 (3.5)	0 (0.0)	
Other	257 (6.8)	16 (5.1)	12 (7.3)	
High-risk women	1735 (46.1)	315 (100.0)	0 (0.0)	
**Glycemic status (reference standard: IADPSG/WHO criteria)**				<0.001
Normal	3764 (100.0)	0 (0.0)	0 (0.0)	
Gestational diabetes mellitus	0 (0.0)	299 (94.9)	160 (96.4)	
Diabetes in pregnancy	0 (0.0)	16 (5.1)	6 (3.6)	
**Events during pregnancy**				
Composite adverse outcome	411 (10.9)*	61 (19.4)	20 (12.0)	<0.001
Preeclampsia	60 (1.6)	5 (1.6)	6 (3.6)	0.14

LGA age infant	338 (9.0)*	49 (15.6)‡	13 (7.8)	<0.001
Shoulder dystocia	5 (0.1)	0 (0.0)	1 (0.6)	0.27
Neonatal hypoglycemia	15 (0.4)*	10 (3.2)	2 (1.2)	<0.001
Ceasarean sectio	744 (19.8)*	84 (26.7)	34 (20.5)	0.01
Preterm delivery (<37 weeks)	196 (5.2)	24 (7.6)	9 (5.4)	0.19
Offspring hospitalization	698 (18.6)	72 (22.9)	42 (25.3)	0.02
Respiratory distress syndrome	173 (4.6)	18 (5.7)	11 (6.6)	0.34
Intrauterine fetal or neonatal death	12 (0.3)	0 (0.0)	1 (0.6)	0.44
SGA infant	368 (9.8)	33 (10.5)	16 (9.6)	NS
Insulin therapy during	0 (0.0)* †	125 (39.7)‡	47 (28.3)	<0.001

Date are n (%) or mean (standard deviation)

HIP: hyperglycemia in pregnancy; LGA: large for gestational age; OGTT: oral glucose tolerance test; SGA: small for gestational age WG: weeks of gestation

Composite adverse outcome: preeclampsia or LGA infant or shoulder dystocia or neonatal hypoglycemia

Symbols insert only if significant ($p<0.05$) after Bonferroni adjustment for multiplicity

*: True negative versus True positive

†: True negative versus False negative

‡: True positive versus False negative

§: yes versus no comparison; NS = non significant

Additional Table S2: Characteristics of the women by true/false positive/negative cases considering Option 1

	True negative cases	True positive cases	False negative cases	p
	n=3764	n=332	n=149	
**OGTT between 22 and 30 WG**				
Fasting plasma glucose (mmol/L)	4.30*(0.36)	5.26‡ (0.44)	4.29 (0.26)	<0.001
1-hour plasma glucose (mmol/L)	6.44*†(1.46)	9.16‡ (2.03)	9.59 (1.28)	<0.001
2-hour plasma glucose (mmol/L)	5.68*†(1.11)	7.89‡ (1.95)	8.60 (1.30)	<0.001
Gestational age when OGTT (WG)	26.21 (1.88)	26.29 (1.92)	26.40 (1.83)	NS
**Characteristics**				
Age (years)	29.99 *† (5.25)	32.34 (5.27)	32.22 (5.60)	<0.001
Preconception body mass index (kg/m^2)	24.18 * (4.37)	26.36 ‡ (5.15)	24.57 (4.46)	<0.001
Preconception hypertension	20 (0.5) *	7 (2.1)	1 (0.7)	0.008
Family history of diabetes	699 (18.6) *	89 (26.8)	36 (24.2)	<0.001
Employment	1677 (44.6)	143 (43.2)	63 (42.3)	NS
Smoking before pregnancy	450 (12.0)	34 (10.2)	9 (6.0)	0.06
Parity	2.02 * (1.18)	2.27 ‡ (1.24)	1.91 (1.14)	<0.001
**Previous pregnancy(ies)**				
History of hyperglycemia in pregnancy				<0.001 §
First child	1589(42.2)	108 (32.5)	72 (48.3)	
No	2089(55.5)	170 (51.2)	65 (43.6)	
Yes	86 (2.3) *†	54 (16.3) ‡	12 (8.1)	
History of macrosomia				<0.001 §
First child	1589(42.2)	108 (32.5)	72 (48.3)	
No	2099(55.8)	206 (62.0)	73 (49.0)	
Yes	76 (2.0) *	18 (5.4)	4 (2.7)	
History of hypertensive disorders				NS §
First pregnancy	1108(29.4)	68 (20.5)	50 (33.6)	
No	2588(68.8)	256 (77.1)	97 (65.1)	
Yes	68 (1.8)	8 (2.4)	2 (1.3)	
History of fetal death				0.03 §
First pregnancy	1108(29.4)	68 (20.5)	50 (33.6)	
No	2612(69.4)	254 (76.5)	98 (65.8)	
Yes	44 (1.2) *	10 (3.0)	1 (0.7)	
**Ethnicity**				<0.001
North African	723 (19.2)	101 (30.5)	42 (28.2)	
European	1369(36.4)	90 (27.2)	50 (33.6)	
Sub-Saharan African	808 (21.5)	69 (20.8)	11 (7.4)	
Indian-Pakistan-Sri Lanka	282 (7.5)	43 (13.0)	17 (11.4)	
Caribbean	263 (7.0)	12 (3.6)	6 (4.0)	
Asian	61 (1.6)	3 (0.9)	8 (5.4)	
Other	257 (6.8)	13 (3.9)	15 (10.1)	
High-risk women	1735 (46.1)	217 (65.4)	98 (65.8)	
**Glycemic status (reference standard: IADPSG/WHO criteria)**				<0.001
Normal	3764(100.0)	0 (0.0)	0 (0.0)	
Gestational diabetes mellitus	0 (0.0)	314 (94.6)	145 (97.3)	
Diabetes in pregnancy	0 (0.0)	18 (5.4)	4 (2.7)	
**Events during pregnancy**				
Composite adverse outcome	411 (10.9)*	66 (19.9) ‡	15 (10.1)	<0.001

Preeclampsia	60 (1.6)	6 (1.8)	5 (3.4)	0.25
LGA age infant	338 (9.0)*	53(16.0) ‡	9 (6.0)	<0.001
Shoulder dystocia	5 (0.1)	0 (0.0)	1 (0.7)	0.26
Neonatal hypoglycemia	15 (0.4) *	11 (3.3)	1 (0.7)	<0.001
Ceasarean sectio	744 (19.8)*	88 (26.5)	30 (20.1)	0.014
Preterm delivery (<37 weeks)	196 (5.2)	24 (7.2)	9 (6.0)	0.28
Offspring hospitalization	698 (18.6)	80 (24.1)	34 (22.8)	0.025
Respiratory distress syndrome	173 (4.6)	18 (5.4)	11 (7.4)	0.25
Intrauterine fetal or neonatal death	12 (0.3)	1 (0.3)	0 (0.0)	1
SGA infant	368 (9.8)	29 (8.7)	20 (13.4)	0.27
Insulin therapy during	0 (0.0) *†	137 (41.3)‡	35 (23.5)	<0.001

Date are n (%) or mean (standard deviation)

HIP: hyperglycemia in pregnancy; LGA: large for gestational age; OGTT: oral glucose tolerance test; SGA: small for gestational age WG: weeks of gestation

Composite adverse outcome: preeclampsia or LGA infant or shoulder dystocia or neonatal hypoglycemia

Symbols insert only if significant ($p<0.05$) after Bonferroni adjustment for multiplicity

*: True negative versus True positive

†: True negative versus False negative

‡: True positive versus False negative

§: yes versus no comparison; NS = non significant

Additional Table S3: Characteristics of the women by true/false positive/negative cases considering Option 1-Sel

	True negative cases	True positive cases	False negative cases	p
	**n=3764**	**n= 217**	**n= 264**	
**OGTT between 22 and 30 WG**				
Fasting plasma glucose (mmol/L)	4.30 *† (0.36)	5.24‡ (0.40)	4.73 (0.63)	<0.001
1-hour plasma glucose (mmol/L)	6.44 *† (1.46)	9.38 (1.96)	9.21 (1.73)	<0.001
2-hour plasma glucose (mmol/L)	5.68 *† (1.11)	8.00 (1.98)	8.20 (1.64)	<0.001
Gestational age when OGTT (WG)	26.21 (1.88)	26.26 (1.88)	26.38 (1.90)	NS
**Characteristics**				
Age (years)	29.99 *† (5.25)	33.16 ‡ (5.35)	31.61 (5.29)	<0.001
Preconception body mass index (kg/m^2)	24.18 * (4.37)	27.57 ‡ (5.59)	24.37 (3.95)	<0.001
Preconception hypertension	20 (0.5) *	5 (2.3)	3 (1.1)	0.008
Family history of diabetes	699 (18.6) *	89 (41.0) ‡	36 (13.6)	<0.001
Employment	1677 (44.6)	88 (40.6)	118 (44.9)	NS
Smoking before pregnancy	450 (12.0)*	14 (6.5)	29 (11.0)	0.046
Parity	2.02 * (1.18)	2.35 ‡ (1.24)	1.99 (1.17)	<0.001
**Previous pregnancy(ies)**				
History of hyperglycemia in pregnancy				<0.001 §
First child	1589 (42.2)	63 (29.0)	117 (44.3)	
No	2089 (55.5)	100 (46.1)	135 (51.1)	
Yes	86 (2.3) *	54 (24.9) ‡	12 (4.5)	
History of macrosomia				<0.001 §
First child	1589 (42.2)	63 (29.0)	117 (44.3)	
No	2099 (55.8)	136 (62.7)	143 (54.2)	
Yes	76 (2.0) *	18 (8.3) ‡	4 (1.5)	
History of hypertensive disorders				NS §
First pregnancy	1108 (29.4)	42 (19.4)	76 (28.8)	
No	2588 (68.8)	170 (78.3)	183 (69.3)	
Yes	68 (1.8)	5 (2.3)	5 (1.9)	
History of fetal death				<0.001 §
First pregnancy	1108 (29.4)	42 (19.4)	76 (28.8)	
No	2612 (69.4)	165 (76.0)	187 (70.8)	
Yes	44 (1.2) *	10 (4.6) ‡	1 (0.4)	
**Ethnicity**				<0.001
North African	723 (19.2)	101 (46.5)	42 (16.0)	
European	1369 (36.4)	35 (16.1)	105 (39.9)	
Sub-Saharan African	808 (21.5)	19 (8.8)	61 (23.2)	
Indian-Pakistan-Sri Lankan	282 (7.5)	43 (19.8)	17 (6.5)	
Caribbean	263 (7.0)	7 (3.2)	11 (4.2)	
Asian	61 (1.6)	3 (1.4)	8 (3.0)	
Other	257 (6.8)	9 (4.1)	19 (7.2)	
High-risk women	1735 (46.1)	217 (100.0)	98 (37.1)	
**Glycemic status (reference standard: IADPSG/WHO criteria)**				<0.001
Normal	3764 (100.0)	0 (0.0)	0 (0.0)	
Gestational diabetes mellitus	0 (0.0)	204 (94.0)	255 (96.6)	
Diabetes in pregnancy	0 (0.0)	13 (6.0)	9 (3.4)	
**Events during pregnancy**				
Composite adverse outcome	411 (10.9) *	53 (24.4) ‡	28 (10.6)	<0.001
Preeclampsia	60 (1.6)	3 (1.4)	8 (3.0)	0.20
LGA age infant	338 (9.0) *	44 (20.3) ‡	18 (6.8)	<0.001
Shoulder dystocia	5 (0.1)	0 (0.0)	1 (0.4)	0.51
Neonatal hypoglycemia	15 (0.4) *	9 (4.1)	3 (1.1)	<0.001

Ceasarean section	744 (19.8)*	62 (28.6)	56 (21.2)	0.006
Preterm delivery (<37 weeks)	196 (5.2)	18 (8.3)	15 (5.7)	0.14
Offspring hospitalization	698 (18.6)†	48 (22.1)	66 (25.0)	0.02
Respiratory distress syndrome	173 (4.6)	10 (4.6)	19 (7.2)	0.16
Intrauterine fetal or neonatal death	12 (0.3)	0 (0.0)	1 (0.4)	0.79
SGA infant	368 (9.8)	20 (9.2)	29 (11.0)	0.77
Insulin therapy during	0 (0.0) *†	98 (45.2) ‡	74 (28.0)	<0.001

Date are n (%) or mean (standard deviation)

HIP: hyperglycemia in pregnancy; LGA: large for gestational age; OGTT: oral glucose tolerance test; SGA: small for gestational age WG: weeks of gestation

Composite adverse outcome: preeclampsia or LGA infant or shoulder dystocia or neonatal hypoglycemia

Symbols insert only if significant ($p<0.05$) after Bonferroni adjustment for multiplicity

*: True negative versus True positive

†: True negative versus False negative

‡: True positive versus False negative

§: yes versus no comparison; NS = non significant

Additional Table S4: Characteristics of the women by true/false positive/negative cases considering Option 2-Sel

	True negative cases	False positive cases	True positive cases	False negative cases	p
	n=3678	n=86	n=229	n=252	
OGTT between 22 and 30 WG					
Fasting plasma glucose (mmol/L)	4.30 (0.36) *†‡	4.46 (0.37) §‖	5.19(0.45) ¶	4.75 (0.64)	<0.001
1-hour plasma glucose (mmol/L)	6.42 (1.46) *†‡	7.48 (1.42) §‖	9.39 (1.94)	9.20 (1.74)	<0.001
2-hour plasma glucose (mmol/L)	5.67 (1.10) *†‡	6.34 (1.20) §‖	8.05 (1.95)	8.17 (1.66)	<0.001
Gestational age when OGTT (WG)	26.21 (1.88) *†‡	26.19 (2.03)	26.26 (1.87)	26.37 (1.92)	NS
Characteristics					
Age (years)	29.93 (5.25) *†‡	32.38 (4.74)	33.23 (5.35) ¶	31.46 (5.26)	<0.001
Preconception body mass index (kg/m^2)	24.15 (4.36) †	25.31 (4.55)	27.41 f (5.57)	24.36 (3.93)	<0.001
Preconception hypertension	19 (0.5)	1 (1.2)	5 (2.2)	3 (1.2)	0.013
Family history of diabetes	671 (18.2) *†	28 (32.6) ‖	94 (41.0) ¶	31 (12.3)	<0.001
Employment	1649 (44.9)	28 (32.6)	93 (40.6)	113 (45.0)	NS
Smoking before pregnancy	447 (12.2) †	3 (3.5)	14 (6.1)	29 (11.5)	0.004
Parity	2.00 (1.17) *†	2.90 (1.05) §‖	2.38 (1.23) ¶	1.95 (1.17)	<0.001
Previous pregnancy(ies)					
History of hyperglycemia in pregnancy					<0.001#
First child	1589 (43.2)	0 (0.0)	63 (27.5)	117 (46.4)	
No	2089 (56.8)	0 (0.0)	100 (43.7)	135 (53.6)	
Yes	0 (0.0) *†	86 (100.0) §‖	66 (28.8) ¶	0 (0.0)	
History of macrosomia					<0.001#
First child	1589 (43.2)	0 (0.0)	63 (27.5)	117 (46.4)	
No	2022 (55.0)	77 (89.5)	148 (64.6)	131 (52.0)	
Yes	67 (1.8) *†	9 (10.5) ‖	18 (7.9) ¶	4 (1.6)	
History of hypertensive disorders					NS#
First pregnancy	1108 (30.1)	0 (0.0)	42 (18.3)	76 (30.2)	
No	2504 (68.1)	84 (97.7)	182 (79.5)	171 (67.9)	
Yes	66 (1.8)	2 (2.3)	5 (2.2)	5 (2.0)	
History of fetal death					0.001#
First pregnancy	1108 (30.1)	0 (0.0)	42 (18.3)	76 (30.2)	
No	2528 (68.7)	84 (97.7)	177 (77.3)	175 (69.4)	
Yes	42 (1.1) †	2 (2.3)	10 (4.4) ¶	1 (0.4)	
Ethnicity					<0.001
North African	694 (18.9)	29 (33.7)	108 (47.2)	35 (13.9)	
European	1353 (36.8)	16 (18.6)	38 (16.6)	102 (40.6)	
Sub-Saharan African	793 (21.6)	15 (17.4)	19 (8.3)	61 (24.3)	
Indian-Pakistan-Sri Lankan	267 (7.3)	15 (17.4)	44 (19.2)	16 (6.4)	
Caribbean	260 (7.1)	3 (3.5)	8 (3.5)	10 (4.0)	

Asian	59 (1.6)	2 (2.3)	3 (1.3)	8 (3.2)	
Other	251 (6.8)	6 (7.0)	9 (3.9)	19 (7.6)	
High-risk women	1649 (44.8)	86 (100.0)	229 (100.0)	86 (34.1)	
Glycaemic status (reference standard : IADPSG/WHO criteria)					<0.001
Normal	3678 (100.0)	86 (100.0)	0 (0.0)	0 (0.0)	
Gestational diabetes mellitus	0 (0.0)	0 (0.0)	216 (94.3)	243 (96.4)	
Diabetes in pregnancy	0 (0.0)	0 (0.0)	13 (5.7)	9 (3.6)	
Events during pregnancy					
Composite adverse outcome	390 (10.6) *†	21 (24.4) ‖	54 (23.6) ¶	27 (10.7)	<0.001
Preeclampsia	59 (1.6)	1 (1.2)	3 (1.3)	8 (3.2)	NS
LGA age infant	318 (8.6) *†	20 (23.3) ‖	45 (19.7) ¶	17 (6.7)	<0.001
Shoulder dystocia	4 (0.1)	1 (1.2)	0 (0.0)	1 (0.4)	0.08
Neonatal hypoglycemia	15 (0.4) †	0 (0.0)	9 (3.9)	3 (1.2)	<0.001
Ceasarean section	721 (19.6) †	23 (26.7)	64 (27.9)	54 (21.4)	0.008
Preterm delivery (<37 weeks)	193 (5.2)	3 (3.5)	18 (7.9)	15 (6.0)	0.30
Offspring hospitalization	677 (18.4) ‡	21 (24.4)	49 (21.4)	65 (25.8)	0.01
Respiratory distress syndrome	166 (4.5)	7 (8.1)	10 (4.4)	19 (7.5)	0.07
Intrauterine fetal or neonatal death	11 (0.3)	1 (1.2)	0 (0.0)	1 (0.4)	0.29
SGA infant	366 (10.0)	2 (2.3)	21 (9.2)	28 (11.1)	0.11
Insulin therapy during	0 (0.0) †‡	0 (0.0) §‖	101 (44.1) ¶	71 (28.2)	<0.001

Data are n (%) or mean (standard deviation)

HIP: hyperglycemia in pregnancy; LGA: large for gestational age; OGTT: oral glucose tolerance test; SGA: small for gestational age WG: weeks of gestation

Composite adverse outcome: preeclampsia or LGA infant or shoulder dystocia or neonatal hypoglycemia

Symbols insert only if significant (p<0.05) after Bonferroni adjustment for multiplicity

*: True negative versus False positive

†: True negative versus True positive

‡: True negative versus False negative

§: False positive versus True positive

||: False positive versus False negative

¶: True positive versus False negative

# yes versus no comparison; NS = non significant

Additional Table S5: Characteristics of the women by true/false positive/negative cases considering Option 3

	True negative cases	True positive cases	False negative cases	p
	n=3764	n=237	n=244	
**OGTT between 22 and 30 WG**				
Fasting plasma glucose (mmol/L)	4.30*† (0.36)	5.43 ‡ (0.40)	4.51 (0.34)	<0.001
1-hour plasma glucose (mmol/L)	6.44 *† (1.46)	8.72 ‡ (2.09)	9.85 (1.34)	<0.001
2-hour plasma glucose (mmol/L)	5.68 *† (1.11)	7.61 ‡ (2.06)	8.60 (1.35)	<0.001
Gestational age when OGTT (WG)	26.21 (1.88)	26.26 (1.91)	26.39 (1.88)	NS
**Characteristics**				
Age (years)	29.99 *† (5.25)	32.24 (5.31)	32.37 (5.44)	<0.001
Preconception body mass index (kg/m^2)	24.18 *† (4.37)	26.63 ‡ (5.41)	25.00 (4.46)	<0.001
Preconception hypertension	20 (0.5)*	5 (2.1)	3 (1.2)	0.01
Family history of diabetes	699 (18.6)*†	63 (26.6)	62 (25.4)	<0.001
Employment	1677 (44.6)	105 (44.5)	101 (41.4)	NS
Smoking before pregnancy	450 (12.0)	21 (8.9)	22 (9.0)	NS
Parity	2.02 * (1.18)	2.34 ‡ (1.27)	1.98 (1.14)	<0.001
**Previous pregnancy(ies)**				
History of hyperglycemia in pregnancy				<0.001 §
First child	1589 (42.2)	72 (30.4)	108 (44.3)	
No	2089 (55.5)	130 (54.9)	105 (43.0)	
Yes	86 (2.3) *†	35 (14.8)	31 (12.7)	
History of macrosomia				<0.001 §
First child	1589 (42.2)	72 (30.4)	108 (44.3)	
No	2099 (55.8)	150 (63.3)	129 (52.9)	
Yes	76 (2.0) *	15 (6.3)	7 (2.9)	
History of hypertensive disorders				0.8 §
First pregnancy	1108 (29.4)	41 (17.3)	77 (31.6)	
No	2588 (68.8)	191 (80.6)	162 (66.4)	
Yes	68 (1.8)	5 (2.1)	5 (2.0)	
History of fetal death				0.051 §
First pregnancy	1108 (29.4)	41 (17.3)	77 (31.6)	
No	2612 (69.4)	189 (79.7)	163 (66.8)	
Yes	44 (1.2)	7 (3.0)	4 (1.6)	
**Ethnicity**				<0.001
North African	723 (19.2)	74 (31.4)	69 (28.3)	
European	1369 (36.4)	61 (25.8)	79 (32.4)	
Sub-Saharan African	808 (21.5)	53 (22.5)	27 (11.1)	
Indian-Pakistan-Sri Lankan	282 (7.5)	30 (12.7)	30 (12.3)	
Caribbean	263 (7.0)	10 (4.2)	8 (3.3)	
Asian	61 (1.6)	3 (1.3)	8 (3.3)	
Other	257 (6.8)	5 (2.1)	23 (9.4)	
High-risk women	1735 (46.1)	157 (66.2)	158 (64.8)	
**Glycemic status (reference standard: IADPSG/WHO criteria)**				<0.001
Normal	3764 (100.0)	0 (0.0)	0 (0.0)	
Gestational diabetes mellitus	0 (0.0)	225 (94.9)	234 (95.9)	
Diabetes in pregnancy	0 (0.0)	12 (5.1)	10 (4.1)	
**Events during pregnancy**				
Composite adverse outcome	411 (10.9) *	47 (19.8)	34 (13.9)	<0.001
Preeclampsia	60 (1.6)	3 (1.3)	8 (3.3)	0.15
LGA age infant	338 (9.0) *	40 (16.9) ‡	22 (9.0)	<0.001
Shoulder dystocia	5 (0.1)	0 (0.0)	1 (0.4)	0.51
Neonatal hypoglycemia	15 (0.4) *†	7 (3.0)	5 (2.0)	<0.001

Ceasarean section	744 (19.8)	62 (26.2)	56 (23.0)	0.034
Preterm delivery (<37 weeks)	196 (5.2)	18 (7.6)	15 (6.1)	0.25
Offspring hospitalization	698 (18.6)*	59 (24.9)	55 (22.5)	0.02
Respiratory distress syndrome	173 (4.6)	15 (6.3)	14 (5.7)	0.36
Intrauterine fetal or neonatal death	12 (0.3)	0 (0.0)	1 (0.4)	0.79
SGA infant	368 (9.8)	22 (9.3)	27 (11.1)	0.77
Insulin therapy during	0 (0.0) *†	98 (41.4) ‡	74 (30.3)	<0.001

Date are n (%) or mean (standard deviation)

HIP: hyperglycemia in pregnancy; LGA: large for gestational age; OGTT: oral glucose tolerance test; SGA: small for gestational age WG: weeks of gestation

Composite adverse outcome: preeclampsia or LGA infant or shoulder dystocia or neonatal hypoglycemia

Symbols insert only if significant (p<0.05) after Bonferroni adjustment for multiplicity

*: True negative versus True positive

†: True negative versus False negative

‡: True positive versus False negative

§: yes versus no comparison; NS = non significant

Additional Table S6: Characteristics of the women by true/false positive/negative cases considering Option 3-Sel

	True negative cases	True positive cases	False negative cases	p
	n= 3764	n=157	n=324	
**OGTT between 22 and 30 WG**				
Fasting plasma glucose (mmol/L)	4.30 *† (0.36)	5.40 ‡ (0.36)	4.75 (0.57)	<0.001
1-hour plasma glucose (mmol/L)	6.44 *† (1.46)	8.97 (2.02)	9.45 (1.72)	<0.001
2-hour plasma glucose (mmol/L)	5.68 *† (1.11)	7.78 ‡ (2.08)	8.27 (1.63)	<0.001
Gestational age when OGTT (WG)	26.21 (1.88)	26.29 (1.84)	26.34 (1.92)	NS
**Characteristics**				
Age (years)	29.99 *† (5.25)	33.08 (5.41)	31.93 (5.31)	<0.001
Preconception body mass index (kg/m^2)	24.18*† (4.37)	27.93 ‡ (5.84)	24.78 (4.20)	<0.001
Preconception hypertension	20 (0.5) *	4 (2.5)	4 (1.2)	0.008
Family history of diabetes	699 (18.6) *	63 (40.1) ‡	62 (19.1)	<0.001
Employment	1677 (44.6)	69 (43.9)	137 (42.4)	NS
Smoking before pregnancy	450 (12.0) *	7 (4.5) ‡	36 (11.1)	0.015
Parity	2.02 * (1.18)	2.39 ‡ (1.25)	2.04 (1.18)	<0.001
**Previous pregnancy(ies)**				
History of hyperglycemia in pregnancy				<0.001 §
First child	1589 (42.2)	44 (28.0)	136 (42.0)	
No	2089 (55.5)	78 (49.7)	157 (48.5)	
Yes	86 (2.3) *†	35 (22.3) ‡	31 (9.6)	
History of macrosomia				<0.001 §
First child	1589 (42.2)	44 (28.0)	136 (42.0)	
No	2099 (55.8)	98 (62.4)	181 (55.9)	
Yes	76 (2.0) *	15 (9.6) ‡	7 (2.2)	
History of hypertensive disorders				0.8 §
First pregnancy	1108 (29.4)	25 (15.9)	93 (28.7)	
No	2588 (68.8)	128 (81.5)	225 (69.4)	
Yes	68 (1.8)	4 (2.5)	6 (1.9)	
History of fetal death				0.008 §
First pregnancy	1108 (29.4)	25 (15.9)	93 (28.7)	
No	2612 (69.4)	125 (79.6)	227 (70.1)	
Yes	44 (1.2) *	7 (4.5)	4 (1.2)	
**Ethnicity**				<0.001
North African	723 (19.2)	74 (47.1)	69 (21.4)	
European	1369 (36.4)	26 (16.6)	114 (35.3)	
Sub-Saharan African	808 (21.5)	15 (9.6)	65 (20.1)	
Indian-Pakistan-Sri Lankan	282 (7.5)	30 (19.1)	30 (9.3)	
Caribbean	263 (7.0)	7 (4.5)	11 (3.4)	
Asian	61 (1.6)	3 (1.9)	8 (2.5)	
Other	257 (6.8)	2 (1.3)	26 (8.0)	
High-risk women	1735 (46.1)	157 (100.0)	158 (48.8)	
**Glycemic status (reference standard: IADPSG/WHO criteria)**				<0.001
Normal	3764 (100.0)	0 (0.0)	0 (0.0)	
Gestational diabetes mellitus	0 (0.0)	148 (94.3)	311 (96.0)	
Diabetes in pregnancy	0 (0.0)	9 (5.7)	13 (4.0)	
**Events during pregnancy**				
Composite adverse outcome	411 (10.9) *	38 (24.2) ‡	43 (13.3)	<0.001
Preeclampsia	60 (1.6)	1 (0.6)	10 (3.1)	0.08
LGA age infant	338 (9.0) *	34 (21.7) ‡	28 (8.6)	<0.001
Shoulder dystocia	5 (0.1)	0 (0.0)	1 (0.3)	0.51
Neonatal hypoglycemia	15	6 (3.8)	6 (1.9)	<0.001

	(0.4) *†			
Ceasarean section	744 (19.8)	43 (27.4)	75 (23.1)	0.03
Preterm delivery (<37 weeks)	196 (5.2)	14 (8.9)	19 (5.9)	0.12
Offspring hospitalization	698 (18.6)	40 (25.5)	74 (22.8)	0.02
Respiratory distress syndrome	173 (4.6)	10 (6.4)	19 (5.9)	0.37
Intrauterine fetal or neonatal death	12 (0.3)	0 (0.0)	1 (0.3)	1
SGA infant	368 (9.8)	15 (9.6)	34 (10.5)	0.91
Insulin therapy during	0 (0.0) *†	69 (43.9) ‡	103 (31.8)	<0.001

Date are n (%) or mean (standard deviation)

HIP: hyperglycemia in pregnancy; LGA: large for gestational age; OGTT: oral glucose tolerance test; SGA: small for gestational age WG: weeks of gestation

Composite adverse outcome: preeclampsia or LGA infant or shoulder dystocia or neonatal hypoglycemia

Symbols insert only if significant (p<0.05) after Bonferroni adjustment for multiplicity

*: True negative versus True positive

†: True negative versus False negative

‡: True positive versus False negative

§: yes versus no comparison; NS = non significant

Additional Figure S1: Flow chart of the study

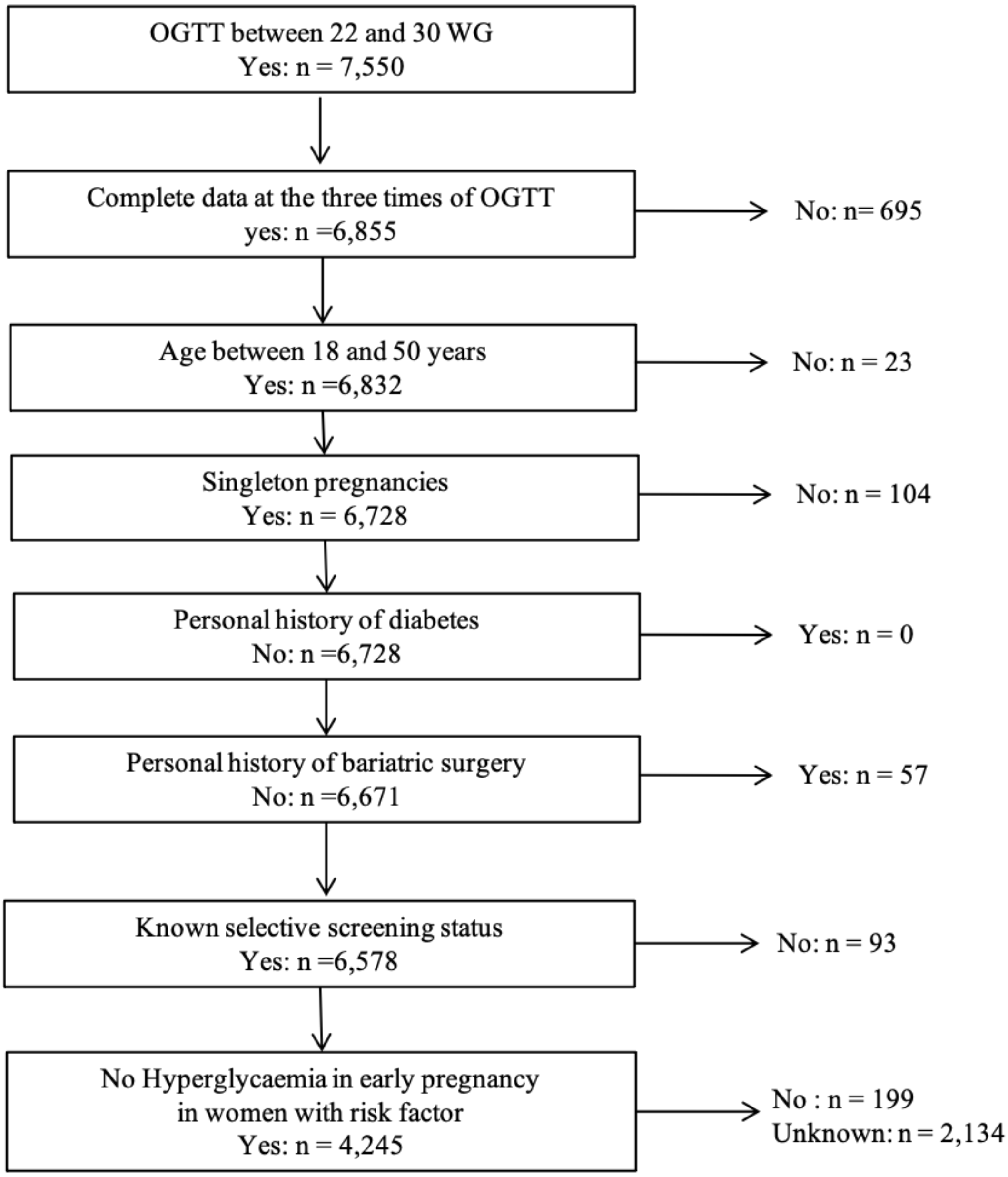

OGTT: 75-g oral glucose tolerance test; WG: weeks of gestation
